# Release Kinetics of Monomers from Dental Composites Containing Fluoride-Doped Calcium Phosphates

**DOI:** 10.3390/pharmaceutics15071948

**Published:** 2023-07-14

**Authors:** Adrián M. Alambiaga-Caravaca, Alicia López-Castellano, Yu Fu Chou, Arlinda Luzi, Juan Manuel Núñez, Avijit Banerjee, María del Mar Jovani Sancho, Salvatore Sauro

**Affiliations:** 1 Department of Pharmacy, Faculty of Health Sciences, Institute of Biomedical Sciences, Cardenal Herrera-CEU University, CEU Universities, C/Santiago Ramón y Cajal, s/n., Alfara del Patriarca, 46115 Valencia, Spain; alacaradr@gmail.com (A.M.A.-C.); alopez@uchceu.es (A.L.-C.); 2 Dental Biomaterials and Minimally Invasive Dentistry, Department of Dentistry, Cardenal Herrera-CEU University, CEU Universities, C/Santiago Ramón y Cajal, s/n., Alfara del Patriarca, 46115 Valencia, Spain; yu.chou2@alumnos.uchceu.es (Y.F.C.); arlinda.luzi@uchceu.es (A.L.); juan.nunez@uchceu.es (J.M.N.); marjovani@uchceu.es (M.d.M.J.S.); 3 Research Centre for Oral & Clinical Translational Sciences, Faculty of Dental, Oral & Craniofacial Sciences, King’s College London, London SE1 9RT, UK; avijit.banerjee@kcl.ac.uk

**Keywords:** dental resin composites, UDMA, HEDA, bis-EMA, residual monomer, HPLC, release kinetics models

## Abstract

This study analyse the type of release kinetic of specific monomers from dental resin composites containing various fluoride-doped calcium phosphates. The release behavior of urethane dimethacrylate (UDMA), ethoxylated bisphenol-A dimethacrylate (bis-EMA) and 1.6-hexanediol ethoxylate diacrylate (HEDA) was evaluated over a period of 35 days. Two tailored calcium phosphates doped with different concentrations of fluoride salts (VS10% and VS20%) were prepared and incorporated in the dimethacrylate matrix at various concentrations to generate a range of experimental composites. The release kinetics were characterized using mathematical models such as zero-order, first-order, Peppas and Higuchi models. The results showed that the first-order model best described the release kinetics. UDMA and HEDA exhibited significant differences in release compared to bis-EMA from day 1, while no significant differences were observed between UDMA and HEDA, except on day 35, when UDMA exhibited a higher release rate than HEDA. When comparing the release of each monomer, VS20-R20% had the highest total release percentage, with 3.10 ± 0.25%, whereas the composite VS10-R5% showed the lowest release percentage, with a total of 1.66 ± 0.08%. The release kinetics were influenced by the composition of the resin composites and the presence of calcium fluoride and sodium fluoride in the calcium phosphate played a role in the maximum amounts of monomer released. In conclusion, the release of monomers from the tested resin composites followed a first-order kinetic behaviour, with an initial rapid release that decreased over time. The composition of the resin monomers and the presence of fluoride salts influenced the release kinetics. The VS10-R5% and VS10-R10% resin composites exhibited the lowest total monomer release, suggesting its potential favourable composition with reduced monomer elution. These findings contribute to understanding the release behavior of dental resin composites and provide insights for the development of resin-based bioactive dental materials.

## 1. Introduction

Dental resin composites represent the main choice to restore carious teeth, thanks to their excellent aesthetics and mechanical properties [1]. However, the National Institute of Health (US-NIH) reported that resin composite restorations have an annual failure rate of up to 15% [2], with an average lifetime of resin composite posterior restorations of less than 6.5 years [3,4]. It has been reported that the main reason for the replacement of dental resin composites is correlated to caries associated with restorations and sealants (CARS—secondary caries) occurring at the tooth–material interface [5,6,7]. This is due to significant uncleansable marginal gap formations, which are then colonised by cariogenic bacteria over time [8].

For example, some specific bioactive glasses were proved to have both an antimicrobial effect [9,10], as well as the ability to remineralize mineral-depleted enamel and dentine [11,12,13]. It seems that the release of calcium and phosphate ions from bioactive compounds can have a noxious effect on some bacteria species and cause neutralisation of the acidic environment in loco [14], thus leading to a local increase in pH that interferes with the metabolism of cariogenic bacteria [15]. Recent studies have reported that experimental dental resin composites doped with bioactive inorganic fillers, may present similar mechanical properties to those of conventional resin composites currently commercialized. [16] Furthermore, novel bioactive glass fillers of different compositions have been recently used to create innovative dental materials for orthodontic splints produced by 3D printing technology [17]. Although recent studies have demonstrated that the inclusion of bioactive glass fillers in resin composites does not jeopardise the degree of monomer conversion [18], there is still little information on the possibility that ions leaching out from such materials can influence the elution of monomers and alter the mechanical properties of such experimental resin composites. Moreover, it is possible that monomers can diffuse through dentine tubules into the pulp in deeper cavities [19,20] and can exhibit cytotoxic effects on oral and dental pulp cells; this issue has raised doubts about their long-term biocompatibility [21].

Currently, there is interest in improving the biocompatibility and the remineralisation ability of resin composites through the incorporation of various bioactive agents, such as fluorine-doped calcium phosphates [12,22]. It would be of great interest to achieve a reduction of the release of monomers when such bioactive fillers are incorporated in resin composites. However, the kinetics of monomer release from resin composites is a complex phenomenon that depends upon multiple factors, including the chemical structure of the monomers, the curing conditions and the presence and the composition of inorganic fillers. All of these factors may play an important role in avoiding excessive release of resin monomers, as well as in maintaining the integrity of the resin composites [23]. Previous studies have shown that the release of monomers from resin composites occurs in a biphasic manner, with a higher initial release followed by a slower sustained release over time [24].

Recently, an experimental calcium phosphate tailored with different concentrations of fluoride salts has been demonstrated to have the ability to convert into biocompatible fluoride-containing apatite-like crystals when immersed in simulated body fluid. Such an innovative bioactive ion-releasing calcium phosphate has been suggested as a promising filler to generate remineralising resin composites and adhesives [22]. However, the effect of such fluorine-doped calcium phosphates incorporated in resin composites as bioactive filler on the release kinetics of monomers has not been tested, and the kinetics of monomer release are not well understood.

**Figure 1:**
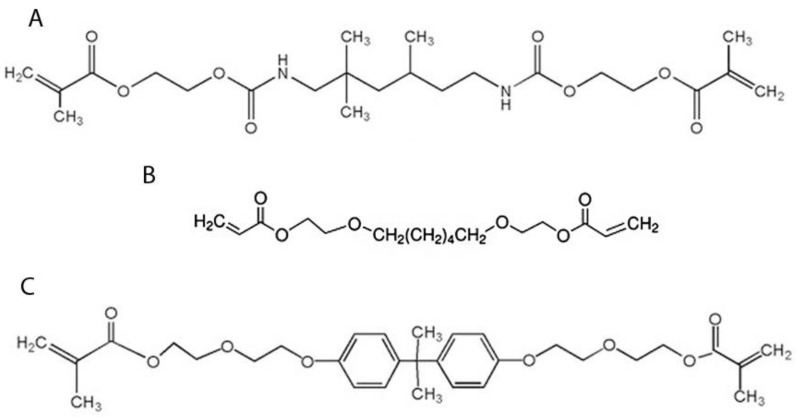
Molecular structures of UDMA (**A**), HEDA (**B**), and bis-EMA (**C**).

Thus, the aim of this study was to investigate the release kinetics of urethane-dimethacrylate (UDMA), 1.6-hexanediol ethoxylate diacrylate (HEDA) and ethoxylated bisphenol-A dimethacrylate (bis-EMA) (Figure 1) from resin composites containing different fillers of calcium phosphate tailored with varying concentrations of fluoride. For this purpose, the total percentage released from the resin composites was evaluated and investigations included whether there were significant differences in the release kinetics of the monomers between the tested resin composites. Moreover, the infinite release tendency of each tested monomer was also investigated in order to determine which composition is the most suitable to reduce the release as much as possible.

## 2. Materials and Methods

### 2.1. Specimen Preparation

Experimental calcium phosphates were formulated by incorporating a constant concentration of calcium hydroxide and different concentrations of fluoride salts, as previously reported in the literature [22]. In brief, a 1:1 molar ratio of beta-tricalcium phosphate (β-TCP) and monocalcium phosphate monohydrate (MCPM) were manually mixed in a glass recipient for 30 s with deionised water (ratio: 1 water/3 powder) for 30 s in the presence of calcium hydroxide (10 wt.%) and calcium fluoride and sodium fluoride (1:1 molar ratio) at two concentrations (10 wt.%. or 20 wt.%, named VS10 and VS20). The experimental vs. fillers were desiccated at 40 °C for 24 h and then dispersed (particle size < 50 μm) at three concentrations (5 vol%, 10 vol% and 20 vol%) in a resin composite made of 55 wt.% UDMA, 20 wt.% bis-EMA and 25% HEDA, (Figure 1). Camphoroquinone and ethyl 4-dimethylaminobenzoate were incorporated at 1 mol%, while trimethylbenzoyl-diphenylphosphine oxide and 2.4 dihydroxybenzophenone were included at 0.5 mol%. Butylated hydroxytoluene at 0.01 wt.% was used as a stabilizer. A nanofiller AEROSIL^®^ R972 (Evonik corp., Essen, Germany), a form of fumed silica with an average diameter of 16 µm, was added at 6 wt.%. All these components, as well as the vs. fillers, were agitated at a rate of 60 rpm in an overhead stirrer mixer (VEVOR SH-2 Magnetic Stirrer, Vevor, Madrid, Spain) at 50 °C in order to obtain a homogeneous resin composite mixture. This final resin composite was used as control resin (VS-R0), while the experimental resins containing different concentrations of the VS10 or VS20 fillers (from 5% to 20%) were (VS10-R5%; VS10-R10%; VS10-R20%; VS20-R5%; VS20-R10%; VS20-R20%). All chemical reagents were purchased from Merck Life Science SLU (Madrid, Spain).

Five specimens were prepared for each tested material using disc-shaped silicon moulds (8 × 2.5 mm). The specimens were positioned over a glass slide that was previously covered with a thin Mylar strip and the tested resins were dispensed directly inside the moulds and covered with a Mylar strip, which was manually pressed using a glass slide. Subsequently, the tested resins were photocured for 40 s each side using a LED light-curing system (>1000 mW/cm^2^, Radii Plus. SDI Ltd., Bayswater, VIC, Australia). The specimens were then removed from the moulds and polished under water irrigation using 1200-grit SiC papers for 30 s.

### 2.2. HPLC Analysis

For the High-performance liquid chromatography (HPLC) analysis, 0.5 mL of each sample was obtained from the storage media at 1, 2, 4, 7, 14, 21, 28 and 35 days and transferred to an HPLC vial for analysis. After each sampling, the extracted volume was replaced with fresh solvent, ensuring that there was no reduction in volume with each sample extracted., and the samples were maintained at 37 °C throughout the whole experiment.

HPLC analysis was performed on a system equipped with a quaternary pump (Waters 1525), an automatic injector fitted with a 50 µL sample loop (Waters 2998 Plus, Madrid, Spain) and a UV/VIS diode-array detector (Waters 2707). The samples were injected on-column using a needle that was previously rinsed using 50% acetonitrile in water. Chromatographic separation was carried out at room temperature (25 ± 2 °C) using a LiquidPurple ODS C18 150 × 4.6 mm reverse-phase column packed with 5 µm silica particles. The mobile phase consisted of a mixture of acetonitrile and pure water in a ratio of 85:15 (*v*/*v*). HPLC grade acetonitrile and ultrapure water were obtained from Análisis Vínicos. S.L. (Tomelloso, Spain). HPLC grade acetonitrile and ultrapure water were obtained from Análisis Vínicos. S.L. (Tomelloso, Spain). The flow rate was set at 1.0 mL/min and the UV detection was set at 215 nm. Retention times were 2.5 min for UDMA, 3.95 for EDA and 5.35 for Bis-EMA. Measurements were at controlled room temperature (25 ± 2 °C). The method used for HPLC analysis was established using modifications of the methods described by Łagocka et al., 2018 [25], Cebe et al., 2015 [26] and by simulating the parameters of the SIELC algorithm.

Standard solutions of UDMA, HEDA and bis-EMA were prepared in absolute ethanol at a concentration of 100 μg/mL for each monomer. Six standard solutions (0.5, 1, 5, 10, 50 and 100 µg/mL) were prepared to create the calibration curve. The analytical validation of the method used in this study was previously validated. Validation results were obtained and these showed linearity (R > 0.999) for each monomer at a concentration range of 0.5–100 μg/mL.

### 2.3. Release Studies

In this study, the accumulated concentrations (µg/mL) of polymers were transformed to percentage release using Equation (1) to accurately account for the percentage of each polymer present. This transformation method provided a more accurate representation of the percentage of each polymer present in the drug delivery system, which is essential for understanding the release kinetics and efficacy of the system.

(1) $$\%=\frac{C\cdot V}{W\cdot P}\cdot 100$$

where C is the accumulated concentration released in the medium (µg/mL), V is the volume (mL) in which the composite was submerged, W is the weight of the composite (µg) and P is the proportion of each resin composite.

In this study, a mathematical modelling strategy was used to analyse the monomer release kinetics from composite materials containing UDMA, HEDA and bis-EMA monomers. The release kinetics of each resin composite were analysed using zero-order, first-order, Korsmeyer–Peppas and Higuchi kinetics to determine which kinetics best fit the data [27,28]. The data plotting and mathematical adjustments were performed employing the Nonlinear Regression–Dynamic Fitting analysis method using SigmaPlot software, version 12.0 (Systat Software Inc., San Jose, CA, USA).

### 2.4. Statistical Analysis

A repeated measures analysis of variance (ANOVA) was performed to assess the differences in polymer release over time among UDMA, HEDA and bis-EMA (R0%). The analysis examined the main effects of polymer type at each time. Post hoc Tukey’s tests were conducted with a Bonferroni correction to account for multiple comparisons. The significance level was α = 0.05.

A two-way robust ANOVA with Bonferroni pairwise comparisons was conducted to compare each R type and determine the statistical differences in the release kinetics between the VS10 and VS20 materials. The factors examined were polymer type (VS10 vs. VS20) and R type (R0, R5, R10 and R20). The analysis controlled for Type I error by applying Bonferroni correction for multiple pairwise comparisons. The significance level was set at α = 0.05.

Independent samples Student’s *t*-tests [29] were performed to compare the mean values of VS10 and VS20 materials at each R type and of each monomer. Bonferroni correction was used for multiple comparisons. The significance level was set at α = 0.05. The assumptions of normality and homogeneity of variances were considered in all of the analyses. All statistical analyses were carried out using IBM SPSS Statistics, Version 28.0. (Armonk, New York, NY, USA).

## 3. Results

The coefficient of determination (R^2^) values of each kinetic model investigated in this study are presented in Table 1. The high R^2^ values indicate that all models exhibited a first-order kinetic behaviour. Table 2 shows the first-order release kinetics constants for the different monomers tested in this study.

The kinetics equation for the first-order release is expressed in Equation (2), where: M_t_ is the amount of substance released at a given time t, M_∞_ represents the equilibrium mass of monomer release that is expected over an extended period beyond the duration of our experimental measurements (35 days), k is the release rate constant and t is the time elapsed since the start of the release.

Mt/M_∞_ = k·t (2)

**Figure 2:**
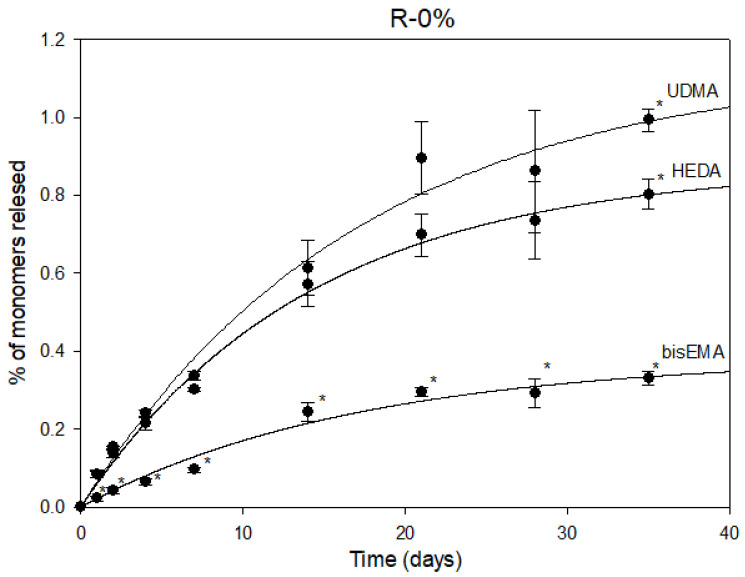
Monomers UDMA, HEDA and bis-EMA in the medium (%) vs. time (days) in release studies for the control filler-free resin composite (R0%). The error bars show the standard deviation of the observed values (n = 6), (* *p* < 0.01).

The first-order kinetics calculated for each monomer are shown in Figure 2. Statistically significant variations in monomer release in the control, filler-free, resin composite were observed between UDMA and HEDA when compared to bis-EMA. In contrast, no statistically significant differences were observed between UDMA and HEDA until day 35, when UDMA exhibited a higher release than HEDA. At day 35, UDMA exhibited the highest release rate (0.99 ± 0.03%), followed by HEDA (0.80 ± 0.04%) and finally, bis-EMA (0.33 ± 0.02%).

**Figure 3:**
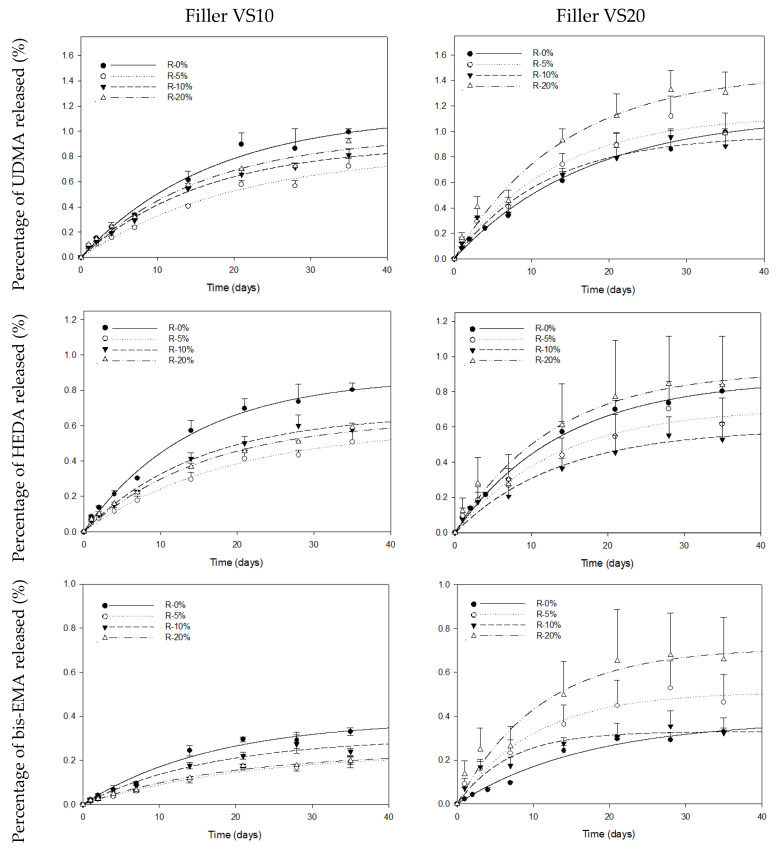
Cumulative release of each monomer (%UDMA, %HEDA and %bis-EMA) versus vs. time (days) for each experimental composite containing the bioactive fillers at different concentrations (R0, R5, R10 and R20). Error bars represent the standard deviation of the observed values (n = 6).

Once the release kinetics of each monomer in the resin composite without calcium fluoride and sodium fluoride were studied, the release kinetics of both VS10 and VS20 at each percentage (R5, R10 and R20) were calculated. Figure 3 shows the release kinetics from the points obtained at each time. The blank is also plotted to compare the effect of the addition of fluoride salts on the release of each monomer. It can be seen in Figure 3 that when fluoride salts are added at 10% (VS10), the release of each monomer tends to decrease, but not at VS20, where the tendency is to maintain or increase the release of the monomers.

From each kinetic release experiment, the percentage at infinity (M_∞_) and the velocity (k), as presented in Table 2, were obtained. Following statistical analysis, it was determined that the VS10-R5% formulation exhibited the lowest total monomer release, amounting to 1.66 ± 0.08% (0.83 ± 0.04%—UDMA, 0.59 ± 0.03%—HEDA and 0.24 ± 0.01%—bis-EMA). Conversely, the VS20-R20% composite displayed the highest total release, measuring 3.10 ± 0.25% (1.47 ± 0.08%—UDMA, 0.92 ± 0.11%—HEDA, and 0.71 ± 0.06%—bis-EMA). In most studies, comparing the release of each monomer within the same vs. system, R-20% demonstrated higher monomer release, except for bis-EMA, where R-10% exhibited the highest release compared to R-5% and R-20% in the VS10 composite. Furthermore, for all monomers formulated in the VS20 resin composites, there was a decrease in release with R-10% compared to R-5%, followed by an increase with R-20%, surpassing the values of R-5%.

**Figure 4:**
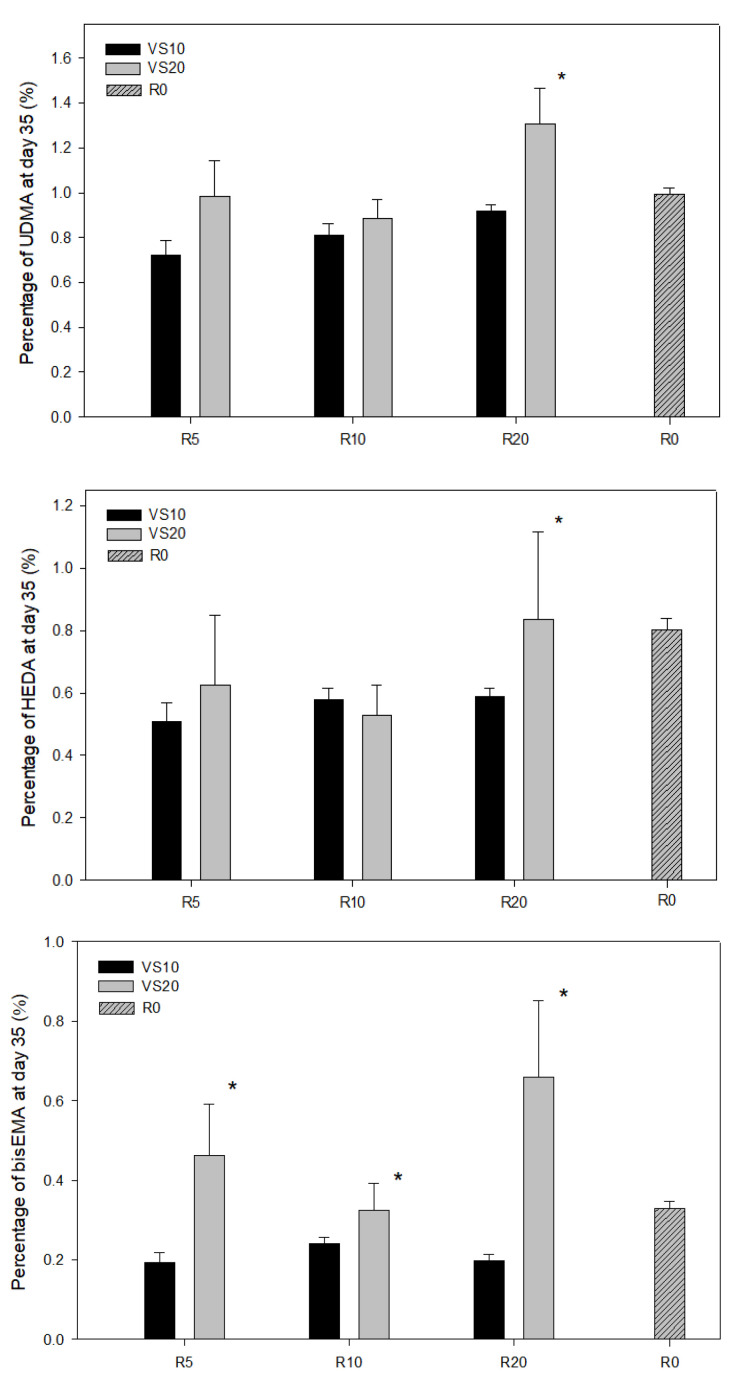
Cumulative % release of each monomer (UDMA, bis-EMA and HEDA) at day 35, comparing VS10 vs. VS20 at each concentration (R 5%, R10% and R20%). The error bars show the standard deviation of the observed values (n = 6), (* *p* < 0.05).

Finally, the results for each polymer were compared on the last day of data collection (day 35) (Figure 4). Although the amounts at infinity showed significant differences between VS10 and VS20 in each and every resin composite formulation, this was not the case at day 35, where only R20 showed statistically significant differences in UDMA and HEDA, with the release of these being higher in VS20 compared to VS10. In contrast, bis-EMA showed statistically significant differences in R5, R10 and R20 between VS10 and VS20 at day 35, with VS20 being superior to VS10. This shows that the differences increase and become more differentiated over longer periods of time.

## 4. Discussion

The composition of dental resin composites, in particular the type and the concentration of resin monomers, plays a fundamental role in the overall biocompatibility and mechanical properties of such products. Indeed, the elution from composite materials of residual monomers, oligomers and other products generated during the degradation processes over time, has been widely studied [30,31,32,33,34]. For instance, it was demonstrated that the elution of such substances from resin composites can induce cytotoxic effects in different types of human and animal cells [3,13]. Moreover, further in vitro studies [31,35] have demonstrated the cytotoxic, genotoxic, mutagenic or estrogenic effects and pulpal and gingival/oral mucosa reactions of some specific monomers released from dental resin composites. The most commonly used monomers to formulate resin composites are bisphenol-A glycidyl methacrylate (Bis-GMA) and triethylene glycol dimethacrylate (TEGDMA). However, a concern has been raised about the toxicity of such monomers when these are released in an unreacted/unpolymerised form [23,36]. Therefore, alternative monomers such as urethane dimethacrylate (UDMA) and bisphenol-A ethoxylated dimethacrylate (bis-EMA) have been recommended to improve the biocompatibility of resin composites. Herewith lies the reason why the primary objective of this investigation was to explore the release kinetics of UDMA, HEDA and bis-EMA from experimental resin composites incorporating bioactive calcium-phosphate fillers doped with varying concentrations of fluoride salts (VS-10 and VS-20). It is important to highlight that this is the first study that analysed the release kinetics of such experimental bioactive composites, as well as the percentage of monomer release. Additionally, a comprehensive examination was conducted to determine the propensity for infinite release of each monomer, with the aim of identifying the best resin composite composition to minimise monomer release. Such fillers (VS-10 and VS-20) incorporated in the experimental resin composites were recently suggested to be potential “bioactive” compounds to incorporate in preventive products such as gels and toothpastes for remineralisation of enamel and dentine, or as inorganic fillers for resin-based adhesives and composites/cements; these latter materials may be able to protect and remineralise the dentine–material interface and extend their clinical performance, especially when applied on caries-affected dentine [22,37].

It is important to consider that resin composites can have important differences in terms of monomer composition, which may influence the viscosity, the mechanical properties and the polymerisation shrinkage [38]. However, the elution of monomers from resin composites can be influenced by several factors, including the extent of the polymerisation reaction and monomer conversion [39]. Moreover, the presence and the type of solvents within the composition may have a significant effect on uncured monomer elution. However, in these experimental resin composites, no solvent was employed.

Several methodologies, including gas-liquid chromatography [40,41] and mass spectrometry [41,42], have been used to quantify the amount of residual monomer eluted from dental materials. Chromatographic methods such as HPLC seem to be a suitable approach for the reliable assessment of resin monomer released from resin composites [41]. The results of the current study provided significant insights into monomer release within the evaluated resin composites. For instance, the analysis of the M_∞_ values (maximum expected release percentage) revealed that the experimental composite VS10-R5% exhibited the lowest total monomer release. This can be attributed to a comparatively lower release rate of these monomers (UDMA: 0.050 ± 0.005 days^−1^, HEDA: 0.053 ± 0.006 days^−1^ and bis-EMA: 0.047 ± 0.006 days^−1^), suggesting a higher capacity of this composite to retain and limit monomer release. Additionally, the release rates correlated with the initial concentration of each monomer within the composition of the tested composites; the monomer having the highest initial percentage (UDMA) showed the highest release rate, while the monomer with the lowest initial percentage (bis-EMA) exhibited the slowest release. This demonstrates the dependence of the release rate on the initial monomer concentration and, consequently, the availability of each monomer.

In the field of substance release, the most commonly employed kinetic models are the zero-order, first-order, Peppas and Higuchi models [43]. These models serve to characterise various aspects of release kinetics. The zero-order model assumes a constant release rate over time, implying that substance release occurs at a constant velocity regardless of the duration of the release process. Thus, the amount of substance released is directly proportional to the elapsed time. The first-order model describes an exponential decrease in the release rate over time. This means that initial release can be fast, but as time progresses, the release rate gradually diminishes. In the first-order kinetic model, the amount of substance released not only depends on time but also on the amount of substance available, that is, the remaining substance to be released. The Peppas model is based on the power law and it is used to characterise non-linear release kinetics. This model is particularly useful when the release of substance from specific materials of drugs does not follow a constant or an exponential rate. It describes more complex and non-linear release patterns that may occur in controlled release systems in the pharmaceutical field. Finally, the Higuchi model is designed to describe linear release from a matrix, such as a polymeric material. Linear release implies that the amount of substance released is directly proportional to the square root of the elapsed time. This model is useful for understanding the controlled release of substances from a stable matrix.

The predominance of the first-order kinetics in this study is attributed to the linear and constant nature of the degradation process. Moreover, this kinetic model postulates that substance release is proportional to the remaining quantity, which could explain its good fit to the experimental data. The release of different monomers occurs at a rate proportional to the concentration of the monomer present in the resin composite. When steady-state is reached in the first-order kinetics, the amount of released substance tends to a constant value as time tends to infinity (concentration at infinity). The concentration at infinity represents the equilibrium achieved in the release system. After a sufficiently long release period, the release rate decreases until the amount of substance released per unit time becomes constant. At this point, the concentration at infinity indicates the maximum amount of substance that can be released from the system. It is important to consider this process in the manufacture and use of dental resin composites to ensure the controlled release of monomers without negatively affecting oral health. The observation that the release of the three monomers studied in this research followed first-order kinetics in the resin composites without salts suggests that this may be the kinetic behaviour of the monomers during release. Moreover, the high degree to which different resin composites with salt compositions exhibited these first-order kinetics and their respective variations suggests that this is the predominant release kinetic behaviour of the monomers.

One of the most significant results obtained in this study was that the VS20-R20% resin composite showed the highest total release, corresponding to higher release rates of each monomer (HEDA: 0.071 ± 0.009 days^−1^, HEDA: 0.078 ± 0.024 days^−1^ and bis-EMA: 0.093 ± 0.023 days^−1^). These results suggest that the chemical structure of each monomer and its interaction with the bioactive fillers may influence the rate of the monomers’ release. Indeed, it was also evident that, except for the VS20-R20%, the remaining resin composites exhibited a reduced release upon incorporation of the vs. fillers compared to control VS-free resin composite. This indicates that a greater amount of fluoride salts within the composition of the bioactive calcium-phosphate filler may jeopardise the stability of the resin composites due to an increase of monomer release.

It is imperative to highlight that fluoride salts are usually incorporated in calcium phosphate to stimulate the formation of fluor-hydroxyapatite and/or fluorapatite [44,45]. Indeed, in dentistry, the remineralisation/demineralisation of dentine and enamel is influenced by pH changes caused by dietary acids intake and/or accumulation of a cariogenic biofilm [46]. Thus, any treatment that would encourage the formation of a more thermodynamically stable apatite such as fluorapatite (FHA) should be prioritised in dental remineralisation [47,48,49]. A recent paper [22] showed that it is possible to produce some tailored fluoride-doped calcium phosphates through a simple method of production, which have the ability to precipitate quickly in the form of apatite-like structures and convert into fluoride-containing apatite after 30 days of immersion in simulated body fluids. However, those fluoride-doped calcium phosphates modified with more than 10% fluoride salts within their composition showed through NMR analysis, a presence of unreacted calcium fluoride, along with the greatest release of fluoride ions over a period of 30 days of storage. It is postulated that the presence of such amounts of fluoride in the VS20 used in the current study to formulate resin composite with the greatest concentration of filler (R20%) may have interfered with the polymer structure and/or with the reactivity of the resin monomers contained in the tested materials. Indeed, previous studies showed that dimethacrylate monomers containing a higher concentration of fluoride exhibited a lower mechanical strength compared to fluoride-free control material due to a decrease of the polymer chain entanglement [50,51]. A further possible explanation is that the presence of fluoride ions generated during water immersion may oxidise part of the C=C bonds, thus inducing a critical degradation effect on the polymer network with consequent increase of release of monomers [52]. Moreover, the presence of fluoride ions may cause a depolymerisation of the matrix–filler interface. Indeed, a previous study [53] advocated three possible mechanisms of interaction between fluoride and resin-based materials: (i) rearrangement of the water monolayer adsorbed on filler where silanols form hydrogen bonds, (ii) hydrolysis of the organosilicon ester group and (iii) disorganization of the siloxane network formed from the condensation of intramolecular silanol groups. All of these mechanisms may have declined the integrity of the particle–matrix interface of the tested composites containing higher concentrations of fluoride salts, thus causing a greater release of monomers. It is important to highlight that F–Na bonding and a concurrent reduction in F–Ca bonding occurred with increased F- content. The dominance of the weaker F–Na linkages resulted in a 40% increase in the diffusion coefficients of fluoride ions [50].

It is important to consider that that the release of UDMA and TEGDMA from a dental resin composite depends on the type of the light-curing system utilised, as well as on the pH and the immersion time in the storage media. Indeed, there may be a significant release of such monomers when halogen lamps are used rather than light-curing systems based on diode lamps. In our study, a diode lamp was employed for the specimen preparation [54].

Moreover, the fillers incorporated within the composition of resin composites may directly influence the elution of monomers [55]. The filler content and its composition, along with the size, and its distribution within the polymeric matrix can influence the both the mechanical properties of the resin composite as well as the elution of the monomers. [55,56]. Furthermore, the filler volume fraction and filler load level of the composites correlate with the material strength and elastic modulus, as well as the fracture toughness of the material [56,57,58].

Another significant result obtained in this study was that the presence of the bioactive filler VS10 up to 20% and VS20 up to 10% within the composition of the experimental resin composites reduced the release of three monomers investigated in this study, compared to the control resin containing no bioactive filler. As previously stated, calcium phosphates doped with fluoride salts at concentration lower than 20 wt.% are able to convert into apatite-like crystals. A previous study demonstrated that the incorporation of calcium phosphate in resin-based materials containing urethane dimethacrylate (UDMA) represents a promising approach to create remineralising materials with a high degree of monomer conversion [54]. The materials tested in this study comprise methacrylates able to polymerise via a free-radical addition reaction, with the free radicals being generated by a photosensitive dye, e.g., camphorquinone (CQ) and co-initiators (e.g., tertiary amines), which are necessary to increase the speed of the polymerisation reaction. It is has been demonstrated that tertiary ionic initiators containing specific atoms such as phosphorus and oxygen may denote the lowest valence number in organic compounds and induce cationic polymerisation through the renewal of the inactive CQ into an original molecule. Furthermore, although these types of molecules are influenced by the photo-polymerisation reaction, the cationic polymerisation activity is most likely due to the electron transfer with the photo-excited molecule (CQ), making it more reactive over time [55]. Indeed, the bioactive micro-fillers used in this study contain such elements and these might have induced a time-extended cationic polymerisation of the uncured monomers within the polymer matrix increasing the degree of conversion overtime in storage. This seems to be due to a physico-chemical Lewis acid–base reaction where resin monomers such as UDMA and HEDA may act as electron donors and the Ca/P fillers represent the electron acceptors [54].

## 5. Conclusions

The findings of this study contribute valuable insights into the monomer release behaviour of resin composites. The VS10-R5% and VS10-R10% resin composites exhibited the lowest total monomer release, suggesting its potential favourable composition with reduced monomer elution. Conversely, the VS20-R20% resin composite demonstrated the highest total release, indicating a higher tendency to release monomers. The presence of fluoride salts in the resin composites generally led to a decrease in monomer release, except for the VS20-R20% composite. The release kinetics of the monomers of the experimental composites, regardless of their additive, follow first-order release kinetics behavior. In other words, the release of the monomers is initially fast but progressively decreases over time as the availability of the monomers to be released diminishes. Thus, both the time and quantity of each monomer present in the dental resin composite influence monomer release. Finally, this study emphasizes the significance of composition, monomer concentration and filler incorporation in influencing the monomer release characteristics of dental resin composites. Such insights are crucial for the development of bioactive dental materials, enhancing their biocompatibility and mechanical performance.

## Figures and Tables

**Table 1:** The R^2^ values for each monomer determined through mathematical fitting using release kinetics models, including zero-order (0), first-order (1), Korsmeyer–Peppas model (P) and Higuchi model (H). The R^2^ values for each monomer were determined by mathematical fitting using release kinetics models of order 0 (0), order 1 (1), Korsmeyer–Peppas model (P) and Higuchi model (H).

	0	1	P	H
UDMA	0.9084	0.9604	0.9493	0.9449
HEDA	0.9047	0.9756	0.9616	0.9610
Bis-EMA	0.8978	0.9639	0.9404	0.9370

**Table 2:** First-order release constants (M_∞_, k) and coefficient of determination (R^2^) for the different monomers tested.

	M_∞_	k	R^2^
**UDMA R0% (VS-free)**	1.14 ± 0.05 ^A^	0.058 ± 0.006	0.9604
**VS10-UDMA-R5%**	**0.83 ± 0.04 ^B^**	0.050 ± 0.005	0.9743
**VS20-UDMA-R5%**	1.13 ± 0.06 ^A^	0.078 ± 0.010	0.9269
**VS10-UDMA-R10%**	0.90 ± 0.02 ^C^	0.063 ± 0.003	0.9897
**VS20-UDMA-R10%**	**0.97 ± 0.04 ^D^**	0.084 ± 0.009	0.945
**VS10-UDMA-R20%**	0.97 ± 0.03 ^C^	0.062 ± 0.004	0.9808
**VS20-UDMA-R20%**	1.47 ± 0.08 ^E^	0.071 ± 0.009	0.9367
**HEDA R0% (VS-free)**	0.87 ± 0.03 ^A^	0.071 ± 0.005	0.9756
**VS10-HEDA-R5%**	**0.59 ± 0.03 ^B^**	0.053 ± 0.006	0.9661
**VS20-HEDA-R5%**	0.71 ± 0.06 ^D^	0.076 ± 0.016	0.8375
**VS10-HEDA-R10%**	0.67 ± 0.02 ^C^	0.067 ± 0.005	0.9793
**VS20-HEDA-R10%**	**0.59 ± 0.04 ^E^**	0.074 ± 0.013	0.8830
**VS10-HEDA-R20%**	0.64 ± 0.01 ^C^	0.063 ± 0.003	0.9887
**VS20-HEDA-R20%**	0.92 ± 0.11 ^A^	0.078 ± 0.024	0.7084
**bisEMA R0%(VS-free)**	0.38 ± 0.02 ^A^	0.058 ± 0.006	0.9639
**VS10-bis-EMA-R5%**	**0.24 ± 0.01 ^B^**	0.047 ± 0.006	0.9639
**VS20-bis-EMA-R5%**	0.51 ± 0.03 ^D^	0.099 ± 0.019	0.8196
**VS10-bis-EMA-R10%**	0.30 ± 0.02 ^C^	0.060 ± 0.008	0.9489
**VS20-bis-EMA-R10%**	**0.33 ± 0.01 ^E^**	0.15 ± 0.02	0.8441
**VS10-bis-EMA-R20%**	**0.23 ± 0.01 ^B^**	0.053 ± 0.004	0.9802
**VS20-bis-EMA-R20%**	0.71 ± 0.06 ^F^	0.093 ± 0.023	0.7385

^A–F^ Statistically significant differences (*p* < 0.05) between the obtained M_∞_ value; values with different superscript indicate that there was a significant difference. Comparisons were made between different fillers (VS10 and VS20) and between R (different vs. concentration) of the same monomer (UDMA, HEDA or bis-EMA). Values are the mean and standard deviation of six replicates. Bold is used to highlight the significant differences.

## Data Availability

Data available on request to the corresponding author.
